# Design and User Evaluation of a Wheelchair Mounted Robotic Assisted Transfer Device

**DOI:** 10.1155/2015/198476

**Published:** 2015-02-22

**Authors:** Garrett G. Grindle, Hongwu Wang, Hervens Jeannis, Emily Teodorski, Rory A. Cooper

**Affiliations:** ^1^Human Engineering Research Laboratories, VA Pittsburgh Healthcare System, 6425 Penn Avenue, Suite 400, Pittsburgh, PA 15232, USA; ^2^Department of Rehabilitation Science and Technology, University of Pittsburgh, 6425 Penn Avenue, Suite 400, Pittsburgh, PA 15232, USA

## Abstract

*Purpose*. The aim of this study is to describe the robotic assisted transfer device (RATD) and an initial focus group evaluation by end users. The purpose of the device is to aid in the transfers of people with disabilities to and from their electric powered wheelchair (EPW) onto other surfaces. The device can be used for both stand-pivot transfers and fully dependent transfers, where the person being transferred is in a sling and weight is fully on the robot. The RATD is fixed to an EPW to allow for its use in community settings. *Method*. A functional prototype of the RATD was designed and fabricated. The prototype was presented to a group of 16 end users and feedback on the device was obtained via a survey and group discussion. *Results*. *Thirteen out of sixteen (83%)* participants agreed that it was important to develop this type of technology. They also indicated that user, caregiver, and robotic controls were important features to be included in the device. *Conclusions*. Participants in this study suggested that they would be accepting the use of robotic technology for transfers and a majority did not feel that they would be embarrassed to use this technology.

## 1. Introduction

The ability of people with mobility impairments to live in their homes and communities with maximal independence often hinges, in part, on their ability to transfer or to be transferred by an assistant. In order to help people with mobility impairments that cannot transfer independently live at home and participate in life's activities, insurance or government agencies may provide for personal attendant care services and in some cases provide stipends for family members providing these services. Further, independent transfers are a common source of upper extremity injuries and joint degeneration that often leads to the need for assistance with transfers over time [1]. Recent research has also shown that many people who can perform independent transfers need assistance when the height differential between transfer surfaces is greater than 75 mm or the gap between surfaces is greater than 150 mm [2]. For people who use power wheelchairs and need human and/or mechanical assistance with transfers, the options are limited. During dependent transfers with a human assistant, there is a high risk of injury (both acute and cumulative) to both the wheelchair user and the assistant, especially over the long term [1].

Between 1973 and 1987, 770 wheelchair-related accidents that led to death were reported to the US Consumer Products Safety Commission. 8.1% of these accidents were caused by falls during transfers [3]. Between 1986 and 1990, there were an estimated 36,000 wheelchair-related accidents in the USA that resulted in a visit to the emergency department. 17% of these accidents were due to falls during transfers [4]. In 2003, more than 100,000 wheelchair-related injuries were treated in US emergency departments, showing an upward trend in the number of injuries over time [5].

When caretakers assist in transferring wheelchair users, there is an additional risk of injury to the caretaker. In one study, of the 48 accidents reported by the 174 participants, 15.5% involved attendants [6]. There were more than 1,325,000 home care workers or clinicians in the United States in 2004. This group is expected to grow by 56% from 2004 to 2014 [7]. Lower back injuries are a major risk for this group, and one estimate found that 10.5% of back injuries in the United States are associated with transferring patients. In one study investigating bed to chair transfers, it was found that healthcare workers experience up to 3500 N of compressive forces during a single transfer [8]. In another study where lifts were implemented in a hospital to assist with patient transfers, it was found that over a 3-year period, there was a 70% decrease in claims cost at the intervention facility. The cost of compensation for injuries at this facility also decreased, with a 241% increase in the comparison facility [9]. Numerous studies have indicated that the US population will continue to age in the coming decades [10, 11] and that the prevalence of disability and impairment has remained high but stable [12].

There are approximately 1.5 million people in the United States who have disabilities that require them to use a wheelchair. One study found that 60% of people reported shoulder pain since beginning their wheelchair use. In comparison, only about 4.7% of the general population report regular shoulder pain [13]. Sitting pivot transfers (SPTs) are ranked among the most strenuous daily tasks of wheelchair users. Repetitions of this task over time can be detrimental to the shoulder and elbow joints of wheelchair users [14].

There are variations in wheelchair users' movements during transfers dependent on their level of injury. When patients transfer themselves from a wheelchair to another surface, most of their weight is initially supported by their trailing upper extremity. As they lose contact with the seat, weight is shifted to the leading arm [15]. During wheelchair transfers, large forces are placed on the shoulder and elbow joints. The leading shoulder encounters higher displacement and velocities than the trailing one [16]. This can cause damage in the leading arm to be accelerated and the onset of pain in this arm to occur sooner.

When wheelchair users are transferred by other people, the biomechanics of the transfer take on a different form. Strain is still placed on the wheelchair users shoulder joints, although it is more evenly distributed across the sagittal plane. There is also an additional factor of strain placed on the lower back of the person assisting with the transfer. One study found that a pivot transfer puts 112 lbs of force onto the clinician assisting with the transfer and raises their risk of developing a lower back disorder to 38.8% [7].

One technique that is used in many healthcare facilities is to move patients using ceiling lifts. In one study where lifts were added to an extended care unit, 71.4% of care staff reported that it became their preferred method of transferring patients and 96% believed that the ceiling lifts made lifting residents easier [17]. While these lifts effectively transfer people without placing as much strain on the caretaker, they are often not used because they are time consuming. In many cases, legislation concerning the implementation of lifts is focused on the caretakers' comfort and safety as opposed to the patients'. In rare cases, these lifts can even subject the patient to bruising or skin tearing. Another major concern when transferring patients using a lift system is that the patient may feel that being moved around in such a manner is undignified [18].

Few high tech devices for transfers are reported in the literature. One such device is the Home Lift, Position, and Rehabilitation (HLPR) chair, developed by researcher at NIST which aims to be able to lift wheelchair users, rotate them, and place them on a toilet, chair, or bed. It has been used to help evaluate how current and future standards could be applied to the HLPR and future robotic transfer devices [19, 20].

The aim of this paper is to describe the design, function, and a focus group evaluation of a novel device for assisting with transfers called the Robotic Assistive Transfer Device (RATD). The purpose of the RATD is to aid in the transfers of people with disabilities to and from their electric powered wheelchair (EPW) onto other surfaces such as a bed, shower bench, toilet, or another chair. The device can be used for both stand-pivot transfers, where the person has some ability to stand and places some weight on the ground, and fully dependent transfers, where the person being transferred is in a sling and weight is fully on the robot. The RATD is fixed to an electric powered wheelchair to allow for its use in both home and community settings. The overarching objective of this study is to engineer solutions to allow people who use power wheelchairs that require assistance (human or mechanical) while transferring to be able to transfer in their own homes, in the homes of friends/family, and in the community at large (e.g., hotels, restaurants, and shopping malls) in a safe, comfortable, efficient, and convenient manner.

The rationale for the conception of this device grew out of the described literature and through previous work on the Personal Mobility and Manipulation Appliance (PerMMA) [21–24]. PerMMA was developed as a test platform for assistive bimanual manipulation and advanced interfaces. While PerMMA and other assistive manipulators were capable of moving small household objects to aid in activities of daily living (ADLs), transfers to preform ADLs like bathing and toileting were not possible with existing hardware. The need for a strong, but less dexterous robotic arm, was perceived.

## 2. Methods

### 2.1. Design

The RATD's design allows for 5 powered degrees of freedom (DOF): two rotary joints, two prismatic joints, and track and carriage subsystem that allows the robot to translate around the seat frame of the wheelchair. When coupled to an EPW, the RATD has 7 overall DOFs. The design of the track and carriage is adapted from previous work on the PerMMA [21] robot and allows the RATD to be used on either side of the EPW seat, greatly increasing its workspace. It also allows the RATD to be stowed behind the seat without adding any width to the EPW when not in use. Proceeding from the carriage to the end effector, the first joint is the shoulder, which rotates internally toward the user or externally away from the user, which is shown in the left and center panel of Figure 3. The shoulder is connected to the proximal segment that contains a prismatic joint. This segment is along the axis of rotation of the shoulder and extends the robots workspace vertically. The proximal segment is connected to the distal segment by an elbow joint, as seen in Figure 2. The distal segment also contains a prismatic joint that allows the end effector to extend away from the elbow.

The robot is powered electromechanically by a combination of planetary gear motors and linear actuators. The carriage is moved around the track using a 24 V, 0.52 A planetary gear motor with a 100 : 1 gear ratio, which is connected to spur gear that propels it along a rack machined in the center of the face of the track. Mechanically, the shoulder joint is a 1.25 inch diameter steel shaft that is fixed to the proximal segment and connected to the carriage with a tapered bearing. It is actuated by a 24 V, 2.2 A planetary gear motor, with a 326 : 1 fixed to the carriage that has a spur gear that pushes another spur gear attached to proximal segment. Proximal and distal segments are identical in construction and are made up of two concentric hexagonal bodies that are able to slide past each other. The bodies are composed of nylon plastic shells created using selective laser sintering (SLS), stainless steel threaded rods, and aluminum end plugs. The combination of elements provides the bodies with strength; the double walled nylon shells provide the compressive strength and the stainless steel threaded rods provide the tensile strength. The aluminum end caps allow threaded rods to be held and tensioned. The concentric bodies are coupled together with a 2500 N linear actuator (Linak, L30) with 250 mm stoke length. Pins inserted through the end plugs and through the clevis ends of the actuator hold the assembly together. An elbow joint connects the proximal and distal segment to each other. A linear actuator (Linak, L30) crosses the joint and powers the elbow to move from 35 degrees to 100 degrees vertically. All three actuators have a spline and nut that prevents them from being back driven. Attached to the end of distal segment is a load cell and handle. Also, attached to the distal segment is a double hook on a swivel, which is used to hang the loops of a transfer sling.

The RATD is equipped with force and position sensors. The position of each joint is tracked using a microcontroller equipped, absolute encoder with digital output (Model A2, US Digital, Vancouver, WA). Two absolute inclinometers with digital output (Model A2T, US Digital, Vancouver, WA) are placed on the base of the wheelchair to determine the angle at which the wheelchair is sitting with respect to gravity. The encoders and inclinometers are able to be daisy-chained to form a network called a Serial Encoder Interface (SEI) bus, which allows data from multiple devices to be transmitted using only four lines. Force sensing is done in two places: at the base of the proximal segment and at the handle. The 6 DOF load cell (Model Omega, ATI-IA, Apex, NC) at the base of the proximal segment can withstand high torque and serves as the primary measurement tool for load on the arm. The second 6 DOF load cell (Model Delta, ATI-IA, Apex, NC) is located between the end of distal segment and the handle. Its primary purpose is to serve as an input device for controlling the arm in conjunction with the handle.

The core electronic components that drive the arm consist of a single board computer (SBC) (Model Cobra, VersaLogic, Tualatin, OR), an analog to digital converter board (Model VCM-DAS-2, VersaLogic, Tualatin, OR), an SEI bus to USB converter (Model SEI-USB, US Digital, Vancouver, WA), and a custom designed relay board, as shown in Figure 4. The SBC provides the programmability, memory storage, and data bus capability to the system. The relay board is used to translate low current digital logic signals from the SBC into high current switching needed to control the motors and linear actuators that power the robot's joints. In addition to receiving computer based signals, the relay board is also capable of accepting inputs from a mechanical switch array to drive each joint. The analog to digital converter is used to digitize the signals from the load cells for use in the control algorithm. Similarly, the SEI to USB converter receives the signals from the encoder network and allows them to be read through a USB port on the SBC to be used in control algorithms. The electronics are powered via a DC-DC converter, which steps wheelchair batteries from 24 v down to ±12 v and 5 v.

**Figure 1:**
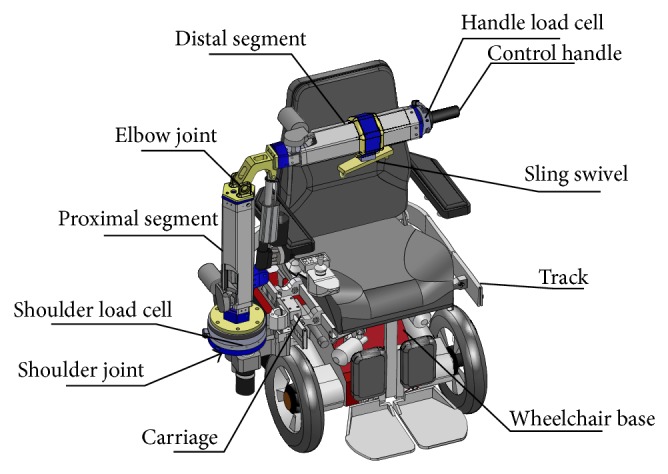
An annotated solid model showing the key mechanical features of the RATD.

**Figure 2:**
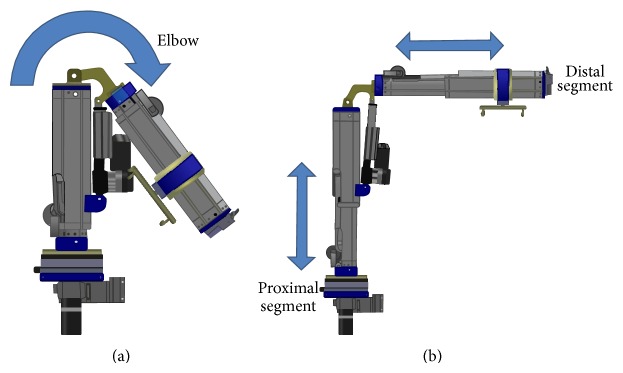
A solid model showing RATD's axis of motion for the shoulder, proximal segment, and distal segment joints.

**Figure 3:**
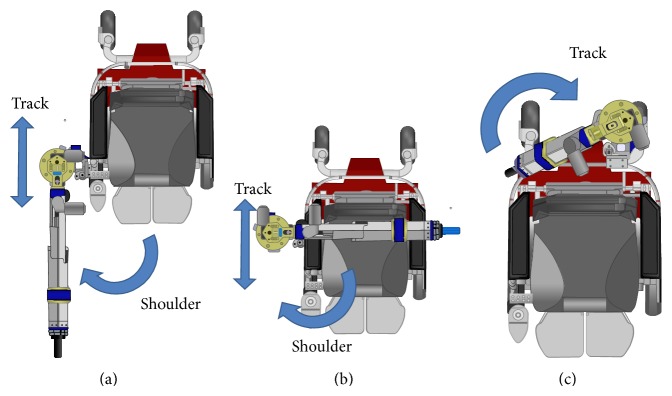
A solid model showing RATD's axis of motion for the shoulder and track joints.

**Figure 4:**
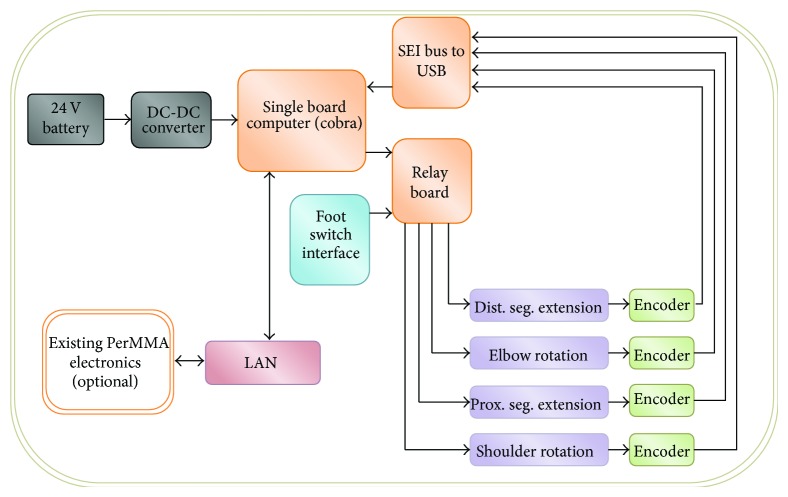
A block diagram describing RATD's motors, sensors, and associated electronics.

The device can be controlled by two different methods by the caregiver: a switch pad or through a force sensing handle method called Direct Interaction. For the switch pad, the carriage and the 4 DOF of the arm are controlled individually with two switches for each DOF, one for each direction of motion. The hardware for the RATD does not have the ability to perform proportional speed control, so motor motion is either on or off. Direct interaction uses a load cell to receive caregiver force inputs through the control handle, visible in Figure 1. The force inputs are mapped to different DOF in an intuitive way to move the RATD. Once one DOF is activated, it locks out the other DOF until the force is removed. The algorithm and force mapping are described in detail by Jeannis et al. [25].

The framework for conceptualizing the safety aspects of RATD is made up of 4 layers. The first of these layers consists of mechanical features, including shrouding of pinch points; rounded edges of metal and plastic surfaces; padding in strategic areas; and compliance, which allows the robot to elastically bend under certain loading conditions. The second layer includes electronic features, including limit switches, hard force limits, hard speed limits, and user initiated emergency stops. The third layer is made up of software features, which allows for the programming of soft force limits, soft speed limits, keep-out zones, and the ability to limit the rate of loading. The fourth layer consists of the human caregiver, who has the ability to observe and make decisions regarding safety.

Stability during transfers is a key safety aspect of the RATD. Force sensors, computational ability, known kinematics, and controlled actuation allow for the use of a dynamically calculated stability boundary that limits the workspace based on payload. A quasistatic mathematical model of the RATD attached to a C500, developed and verified by Wang et al. [26], accounts for the COM of the C500 and the payload on the RATD and determines the safe workspace boundary for that payload. An additional algorithm tracks the kinematics of the RATD and can prevent it from moving into a region that would cause a tip-over.

A demonstration protocol was created to determine if the RATD could perform transfers. A 185-pound extrication dummy known as “Survivor” (Dummies Unlimited, Inc., Pomona, CA) was used as a surrogate for the wheelchair user. Four surfaces were identified: a mat table, a shower bench, a toilet in a restricted space, and soft chair with arms. The transfers were performed to and from each object and with both the key pad and Direct Interaction. The tasks were evaluated as pass-fail, the transfer was considered complete when Survivor was situated in the middle of the surface, and the time period was unlimited.

### 2.2. Focus Group Protocol

In order to obtain qualitative feedback regarding the concept for the RATD, a focus group was conducted. A convenience sample of 18 participants was recruited at the 2011 National Veteran Wheelchair Games in Pittsburgh, PA. In order to participate, participants had to report that they used some type of wheeled mobility as primary means of mobility. This was to include people who normally transferred dependently and independently to better determine who might use the device and in what context. After obtaining written informed consent, each person was asked to fill out a presurvey that asked questions regarding their demographic information, types of assistive technology (AT) they used, and their satisfaction with that AT. Following the presurvey, the participants were shown a live demonstration of the RATD and an explanation of the device by the design team. Participants were given the opportunity to ask the design team questions. A moderator, who was not involved with the design of the device, then led a group discussion of the device. The moderator probed the group as to what features of the device they like or disliked, what features they would like to see added, and, if they would use the device, in what context would they use the RATD. The conversation was recorded using a digital recorder. Following the group discussion, the participants were asked to fill out a postsurvey that asked questions related to the RATD and gave an additional opportunity to make general comments about the device. The postsurvey contained a set of questions in which the participants were given a design feature related to the RATD and were asked to rate on a 7-point Likert scale if the feature would make them less likely to want the device (1) or more likely to want the device (7). It also contained a second set of questions in which the participants were given a statement and asked to what extent they disagreed (1) or agreed (7) with the statement on a 7-point Likert scale. The focus group lasted about an hour and a half, from start to finish.

For the purpose of analysis, the Likert scale responses were collapsed. For the question on product features, responses of 1 and 2 were categorized as “less likely,” 3, 4, and 5 as neutral, and 6 and 7 as “more likely.” For the statement questions, responses of 1 and 2 were categorized as “disagree,” 3, 4, and 5 as “neutral,” and 6 and 7 as “agree.” The responses were compiled using Excel and a descriptive analysis of the data was completed using SPSS.

## 3. Results

**Figure 5:**
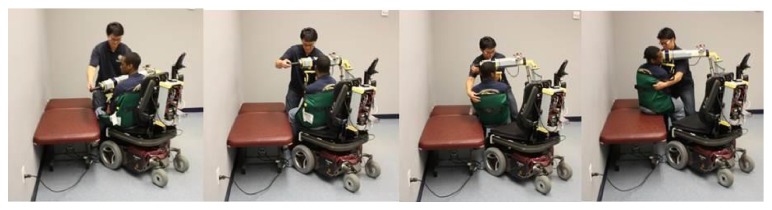
A sequence of photographs of the RATD being used to transfer a person from an electric powered wheelchair to a mat table, by a caregiver.

Figure 5 shows a sequence of photographs that demonstrate a caregiver using the RATD to transfer a wheelchair user from an EPW to a mat table. The RATD was able to perform all the transfers during the demonstration protocol.

**Figure 6:**
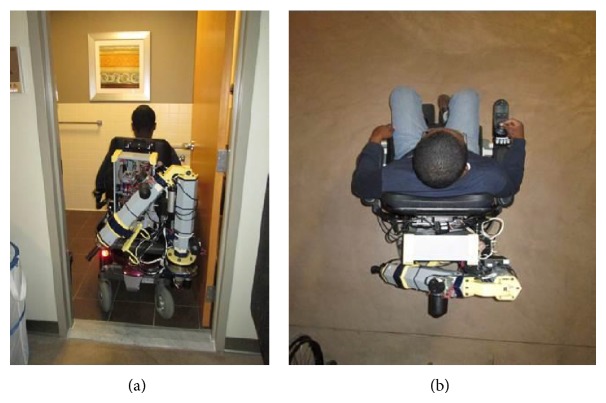
The RATD in its stowed position. (a) It is shown while passing through a doorway and (b) it is shown from above.

In the stowed position, the RATD fits within the footprint of the C500 and can fit through any doorway that a C500 without an RATD can fit through, as shown in Figure 6.

Of the 18 participants recruited, 16 finished the study and an analysis was performed using data from only the participants that finished the study. The group consisted of 11 males and 5 females, all of whom were Veterans. The participants were an average of 20 ± 13 years post onset of disability. 8 participants used manual chairs and 8 participants used powered mobility. The types of disabilities represented in this study are given in Table 1.

**Figure 7:**
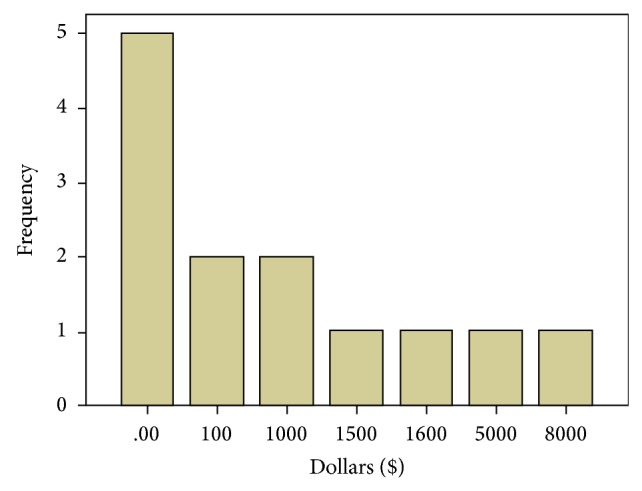
Histogram of how much the participants were willing to pay out of pocket for RATD in dollars.

When asked “How much money out of pocket would you pay for the RATD?” the participants responded with an average of $1407.69 ± 2416.42 and a range of $0–8,000. A histogram showing the distribution is given in Figure 7. Three participants declined to answer this question. When asked if having a transfer device attached to a wheelchair would make them more or less likely to want it, 1 (6%) responded with less likely, 9 (56%) responded with no difference, and 6 (38%) responded with more likely. When asked if having a transfer device controlled by a caregiver would make them more or less likely to want it, 1 (6%) responded with less likely, 5 (31%) responded with no difference, and 10 (63%) responded with more likely. When asked if having a transfer device controlled by a computer program would make them more or less likely to want it, 1 (7%) responded with less likely, 6 (43%) responded with no difference, and 7 (50%) responded with more likely, with two participants declining to answer the question. When asked if having a transfer device controlled by the user would make them more or less likely to want it, 1 (6%) responded with less likely, 5 (31%) responded with no difference, and 10 (63%) responded with more likely. A summary of these responses is given in Table 2.

The results of the survey pertaining to agreement with a particular statement are summarized in Table 3.

Three notable themes were brought up during the group discussion. The first was that the device would be especially good for travel. The RATD would minimize the amount of equipment that would need to be transported and that it would be easier to adapt to bathrooms that have less than ideal accessibility. The second is that the device should also be available with a user interface, so that persons with a disability could transfer themselves without a caregiver. It was noted in the discussion that the RATD could provide a range of transfer assistance from dynamically adjustable grab bars, through stand-pivot transfers, to fully dependent sling transfers. The participants suggested that those needing less assistance would likely want to control the RATD themselves. Lastly, the participants indicated dissatisfaction with current sling technology for dependent transfers and that the RATD might open up new possibilities for improved slings or harnesses for both dependent and stand-pivot transfers.

## 4. Discussion

### 4.1. Design

During the demonstration protocol the RATD was able to be used to transfer Survivor from the wheelchair to all the surfaces and back. The initial position of the wheelchair was critical to being able to complete the transfer. This suggests that either the RATD workspace needs to be increased or additional aids are needed to locate the EPW efficiently near the surface. While no strict scientific evaluation was performed, Survivor behaved well as a transfer surrogate. In the future, if a transfer surrogate can be validated and standardized, it will greatly aid algorithm development and progress in the field.

The number and types of degrees of freedom were selected deliberately when creating the RATD and much information was drawn from prior work with the PerMMA project. While working with PerMMA it was observed that humans can control prismatic joints better than rotational joints. Since human control is an important aspect of the RATD concept, effort was made to include multiple prismatic joints to reduce cognitive load on the caregiver. The arm portion of the system has 4 DOFs, which may seem counterintuitive, since this leaves the arm highly constrained. However, unlike most robotic arms, which are designed for finer manipulation, the task of moving a person is gross movement and the typical wrist-like DOFs in other robotic arms are not necessary. Fewer DOFs reduce control complexity and save physical space, which is paramount on a mobile device.

### 4.2. Focus Group

The responses to the survey yielded some notable results. In regard to product feature Table 2 A1, the majority of the participants were either neutral or supportive of the idea of having a transfer device attached to a power wheelchair, with only small minority objecting to this idea. This suggests that there is not a categorical bias against having a combination mobility and transfer device. Product features Table 2 A2, A3, and A4 were aimed at determining what types of controls the participants were comfortable with, especially contrasting computer/robotic control of the device versus the more traditional user or caregiver control that is used on typical assistive devices. Given that the responses to all three types of controls were similar, this suggests that people are not categorically biased against computer programs controlling their device and that several control methods are likely necessary to accommodate different people and the different contexts for which they might use a transfer device.

The responses to statements Table 3 B3, B4, and B5 also suggest that the participants would be accepting this robot technology. With statement Table 3 B3, the majority of the participants agreed that they would be able to learn how to use the RATD, which is contrary to the common perception of robots as complicated. Possible explanations for this might be that people are growing more comfortable with high tech devices or that the limited number of inputs and prismatic joints make the RATD more manageable to operate. With statement Table 3 B4, the majority of participants suggested that they would not be anxious or would have neutral feelings when using the RATD, which again may be contrary to common perceptions of robots. This may reflect that participants are at least willing to trust a robotic transfer device but may be cautious while doing so. With statement B5, a strong majority of participants indicated that they would not be embarrassed to use the RATD, suggesting that the participants do not perceive any negative social bias toward the device.

The response to statement Table 3 B6 suggests a possible weakness of the RATD. The group was split on whether seeking additional caregiver help would be easier than using the device. While evidence strongly indicates that transferring without properly used equipment is dangerous, this response suggests that humans are still considered an alternative to transfer technology by people with disabilities. Until transfer technology overcomes the speed and adaptability of humans, this perception will likely persist and is a key challenge for developers of transfer devices.

In order to better interpret the results, some discussion of the participants is warranted. While all the participants used wheeled mobility, some had the ability to independently transfer some needed partial assistance, and others were completely dependent on caregivers for transfers. For survey questions such as Table 3 B1, the participants' ability to transfer likely influenced their response. Future work should focus specifically on people who need some sort of assistance for transfers and in what context they would use the device. However, a strong majority agreed with statement Table 3 B7 that a transfer device with RATD capabilities was important to develop. This suggests that while some of the participants might not have a current need for the device, they could see that others might be able to benefit from it or that they might be able to benefit from it as their abilities change in the future. In regard to how much the user would be willing to pay out of pocket, most of the participants indicated that they would pay little or no money out of pocket for the device. This suggests that the participants expect 3rd-party payers to fund the device.

As noted in the methods, a convenience sample was used and this may have led to participants having a preferential bias toward new technology and may have given more positive answers than the general population. Also, the data was collected from individuals at a recreational event and this again may have resulted in a biased sample toward people who are active and may have excluded those who are not able to be. Including the less active might have resulted in identifying other transfer-related barriers. Lastly, while precautions were taken to minimize the role of the design team, they were present in the room for the presentation and discussion. The participants' perception of the design team may have influenced the participants' attitude toward the device in positive or negative way.

It should be noted that this study has several limitations including small sample size, a relatively homogenous sample, and the inherent limitations of qualitative data. In regard to the homogeneous sample, all participants were Veterans, were predominately male, and all had acquired conditions. However, the population that may benefit from this device is likely very heterogeneous. Future design development should focus on improving controls for caregivers; creating user controls; further refinement of algorithms for tip-over stability to include nonlevel surfaces; and optimizing the device for cost, size, aesthetics, and reliability. Future experimental studies should focus on comparing the device to existing technology and the role of caregivers.

## Figures and Tables

**Table 1:** The disability and frequency of participants.

		Count
Disability	SCI	9
	Amputation	1
	MS	2
	TBI	1
	TBI and amputation	1
	Back injury	1
	Hemiparalysis	1

**Table 2:** The responses to the survey questions related to product features.

Product feature	Responses (*n* = 16 unless noted)		
	Less likely to want it	No difference	More likely to want it
(A1) A transfer device attached to a power wheelchair.	1 (6%)	9 (56%)	6 (38%)
(A2) A transfer that can be controlled by a caregiver.	1 (6%)	5 (31%)	10 (63%)
(A3) A transfer device that can be controlled by a computer program. (*n* = 14)	1 (7%)	6 (43%)	7 (50%)
(A4) A transfer device that can be controlled by the user.	1 (6%)	5 (31%)	10 (63%)

**Table 3:** The responses to the survey questions related to agreement or disagreement with a statement.

Statement	Responses (*n* = 16 unless noted)		
	Disagree	Neutral	Agree
(B1) I would choose to use the RATD.	4 (25%)	9 (56%)	3 (19%)
(B2) Using the RATD would make my life easier.	3 (19%)	9 (56%)	4 (25%)
(B3) Leaning to use the RATD would be easy for me.	1 (6%)	6 (38%)	9 (56%)
(B4) I would be anxious about using the RATD.	6 (38%)	8 (50%)	2 (13%)
(B5) It would be embarrassing to be seen using the RATD. (*n* = 15)	11 (73%)	3 (20%)	1 (7%)
(B6) It would be easier to just get another person to help rather than use the RATD.	6 (38%)	5 (31%)	5 (31%)
(B7) It is important that we develop technology that can do this.	0 (0%)	3 (19%)	13 (81%)
